# Molecular Evolution of GII-4 Norovirus Strains

**DOI:** 10.1371/journal.pone.0041625

**Published:** 2012-07-26

**Authors:** Katherina Zakikhany, David J. Allen, David Brown, Miren Iturriza-Gómara

**Affiliations:** 1 Virus Reference Department, Health Protection Agency Microbiology Services, London, United Kingdom; 2 The European Programme for Public Health Microbiology Training (EUPHEM), European Centre for Disease Prevention and Control (ECDC), Stockholm, Sweden; National Institute of Allergy and Infectious Diseases, United States of America

## Abstract

**Background:**

Human Noroviruses (NoV) are the major cause of acute nonbacterial gastroenteritis and the leading cause of outbreaks of gastroenteritis worldwide. Genotype II-4 (GII-4) NoV has been shown to spread rapidly and is the most commonly detected strain worldwide, particularly in association with outbreaks. Previously, we have shown that circulating GII-4 NoV strains exist as populations of selectively neutral variants, and that the emergence of epidemic GII-4 NoV strains correlated with mutations in at least two key sites (Sites A and B) within the P2 domain of the surface exposed major capsid protein (VP1).

**Methodology:**

We developed a rapid pyrosequencing method for screening of the two Sites A and B and a homology based modelling system was used to predict the effects of amino acid substitutions at these sites on the antigenic properties of the virus (defined as surface motif types).

**Principle Finding/Conclusion:**

Here, we describe the characterisation of amino acid diversity at Sites A and B for 1062 GII-4 NoV strains from clinical specimen associated with outbreak of gastroenteritis (2000–2011) and 250 GII-4 NoV sequences from Genbank. Our data identified a high diversity of different Site A and B site combinations at amino acid level and amino acid diversity was higher at Site B than Site A. Site A motifs could be grouped into 3 clusters based on similar surface motif types. We predict that Site A is a major epitope on the virus surface, responsible for defining the antigenic profile, and a more subtle role for Site B, maintaining minor antigenic variation within the virus population.

## Introduction

Norovirus (NoV) is the leading cause of acute viral gastroenteritis in humans worldwide, affecting people of all ages [1], [2]. A study into community cases of infectious intestinal disease in the UK estimated that 3 million episodes of gastroenteritis each year are attributable to NoV [1]. Outbreaks of NoV gastroenteritis are often reported in semi-closed environments (such as schools, the military or cruise ships), but have the greatest impact and occur with highest frequency in health care settings, particularly hospitals and nursing homes. In 2009, over 2300 outbreaks of NoV gastroenteritis in hospitals in England affected over 24,000 patients (HPA, http://www.hpa-bioinformatics.org.uk/noroOBK/).

Gastroenteritis associated with NoV is usually mild and self-limiting but can have a serious impact on high risk groups such as elderly or immunocompromised people, causing prolonged morbidity and contribution to excess mortality [3], [4], [5]. Typically the illness presents as vomiting and/or diarrhoea, with onset 12–48 hours after infection, and symptoms generally last for 12–48 hours.

The Norovirus genus is one of four genera in the *Caliciviridae* family, which are small, non-enveloped, single-stranded (ss)RNA viruses. The NoV genome is ∼7.5 kb and is organised into three open reading frames (ORF): ORF1 encodes a polyprotein that is cleaved into smaller non-structural proteins; ORF2 and ORF3 encode the major (VP1) and minor (VP2) capsid proteins, respectively.

There are 5 NoV genogroups (GI–GV), based on the VP1 sequence, and these are further subdivided into multiple genotypes [6]. The majority of human NoV strains are found in genogroups GI and GII, and genogroup II-genotype 4 (GII-4) strains and are the most commonly detected strains worldwide and are the most frequent NoV associated with outbreaks in healthcare settings [2].

**Figure 1:**
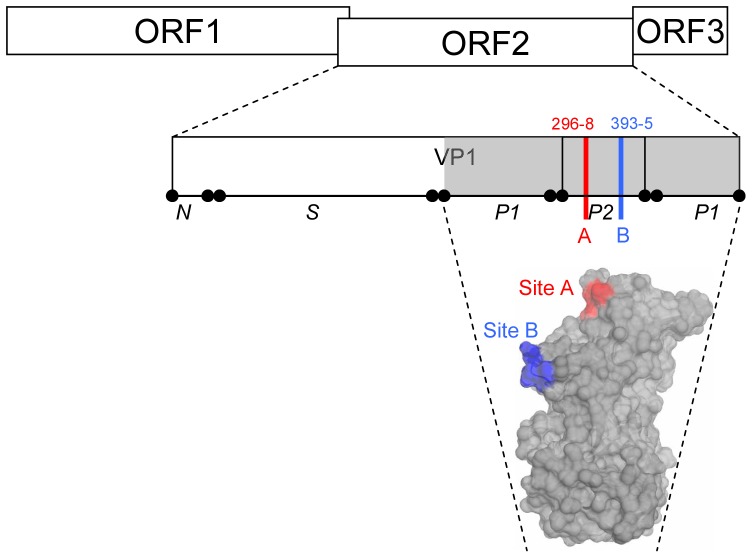
Location of Site A and Site B in the GII-4 NoV P2 domain. The NoV genome has three open reading frames (ORF1, 2, 3) and the major capsid protein (VP1) is encoded by ORF2. The VP1 protein has three main domains: an N-terminal domain (N), the highly conserved shell domain (S), and the protruding domain (P) which forms surface exposed spikes on the virus surface, which is further subdivided into hypervariable P2 domain (P2) and the more conserved P1 domain (P1). Previously, we identified two antigenic sites in the hypervariable P2 domain of the GII-4 NoV capsid protein [15]. Site A (shown in red) is comprised of consecutive amino acid residues 296–298. Site B (shown in blue) is comprised of continuous amino acid residues 393–395. Amino acid position numbering relative to prototype strain Lordsdale/1993/UK (Accession Number: X86557), residues mapped onto the surface of GII-4 NoV strain VA387 P-domain crystal structure (PDB Number 2OBS) described by Cao et al [22].

The ability of NoV to cause large and widespread outbreaks is associated with several factors. The infectious dose of NoV is predicted to be very low [7], and it is transmitted via a faecal-oral route through direct contact, aerosol droplets or fomites. The immunity generated following NoV infection wanes rapidly (approximately 6 months), and any cross-protective immunity is likely to be limited [8].

A number of factors have been identified that may influence the susceptibility in the population to NoV infection. Early population studies suggested that a host genetic factor may have a role in determining individual host susceptibility to NoV infection [9], [10]. Two host factors implicated in the susceptibility to NoV are histo-blood group antigens (HBGAs) and host immunity.

The HBGAs are terminal carbohydrates, whose stepwise synthesis is catalysed by a family of glycosyltransferase enzymes. The expression of HBGAs differs among tissues: ABH and Lewis antigens are expressed on the epithelia of tissues that are in contact with the external environment, such as the respiratory and gastrointestinal tracts. HBGA molecules are also secreted into the saliva of some individuals. As HBGAs are expressed differently across the population, based on an individual’s genetic profile, these have been postulated as susceptibility factors, and it has been proposed that these are utilised as attachment molecules by NoV [11]. Studies suggest that different NoV genotypes have different HBGA recognition profiles [11], [12] and that within a single genotype HBGA recognition profiles can change over time [13], [14].

The interactions between virus and host immune system is likely to be another key factor influencing the population susceptibility to NoV. The genetic diversity observed among GII-4 NoV strains is likely to be, at least in part, driven by selective immunological pressure at a population level [14], [15], [16]. Genetic drift has been shown to lead to the generation of diversity among GII-4 NoV strains, and several positions – mostly located within the hypervariable region (P2 domain) – have been described as potential epitopes which are likely to be subject to immune selection [14], [15], [16].

Previously, we have shown that GII-4 NoV strains exist as populations of selectively neutral variants, and that periodically, the emergence of novel GII-4 NoV strains associated with epidemic waves of gastroenteritis correlated with mutation in at least two key sites within the P2 domain [15], [17]. These two motifs (A and B) in the P2 domain, each three amino acids in length, localise to surface exposed loops on the capsid surface and function as GII-4 variant-specific epitopes (Figure 1) [18].

In this study we aim to further characterise the diversity at hotspot Sites A and B among circulating GII-4 NoV strains. We developed a rapid pyrosequencing method for screening of the two sites, which was used for monitoring sequence diversity of Site A and Site B for a panel of 1062 GII-4 NoV clinical specimens.

**Figure 2:**
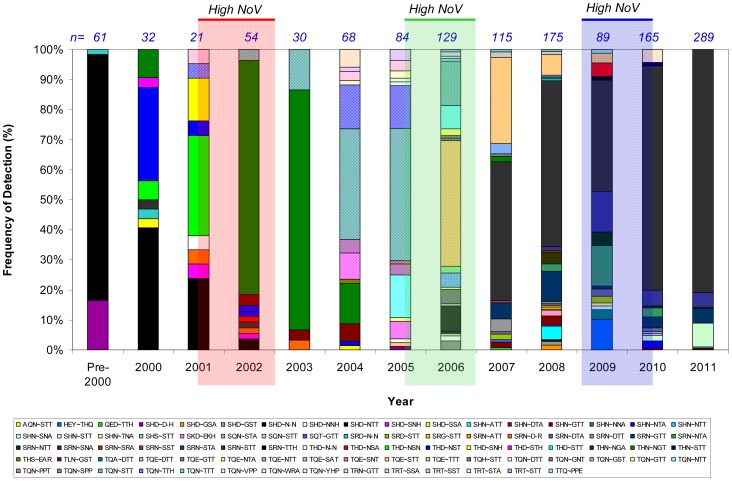
Detection frequencies of Site A/B motif combinations. In total, we detected 23 Site A and 41 Site B motifs in 82 combinations during the analysis period. In 2001–2002 the motif combination [SHD∼N–N] (black bar) was replaced by [THN∼NGT] (dark green bar). This motif combination dominated until 2004 when it was replaced by [TQN∼STT] (hatched black & blue bar), and in 2006, this motif was succeeded by motif combination [TQE∼STT] (red & orange hatched bar). By 2008, [TQE∼STT] had been replaced by [SRN∼STT] (grey bar). The total numbers of strains per year are given above the columns. Shaded columns highlight periods of high NoV activity in 2001/2002 (red), 2005/2006 (green) and 2009/2010 (blue). Amino acid motifs designated by standard IUPAC single-letter amino acid code. For more information on strain replacement events, see Figure S1.

## Methods

### Clinical Samples

A total of 1062 faecal samples representative of NoV-confirmed outbreaks occurring in England between 2000–2011 were selected from an archive held at the Enteric Virus Unit, Health Protection Agency, London UK. Clinical samples were prepared as 10% suspensions in a balanced salt solution (Modified Eagles Medium, Gibco, Life Technologies, Paisley, UK).

**Figure 3:**
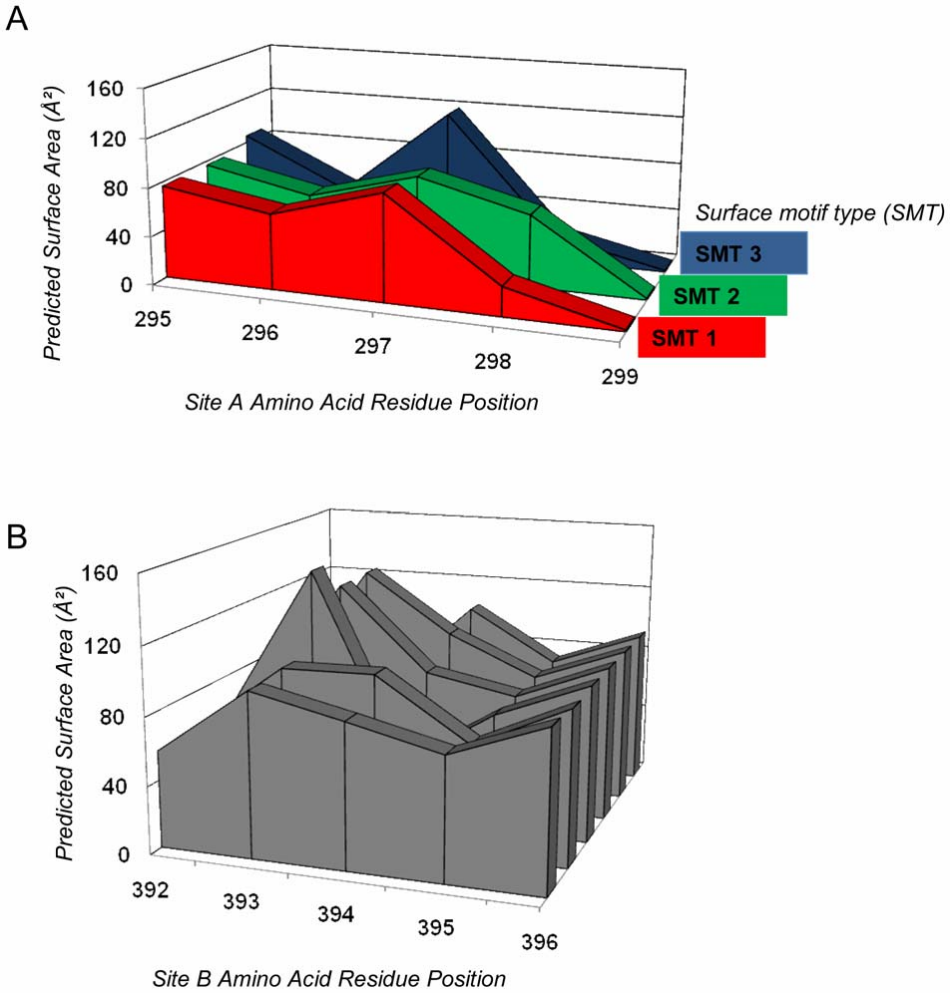
Predicted surface motif types (SMT) of (A) Site A motifs, and (B) Site B motifs. Predicted surface area profiles were generated and the surface area values (Å^2^) of key amino acid residues were plot graphically. Similar profiles were grouped together as surface motif types. (A) Predicted surface motif types for Site A (amino acid residues 296–298) revealed three clusters of profile types: surface motif type 1 (red) detected 2000–2011; surface motif type 2 (green) detected 2004–2009; surface motif type 3 (blue) detected 2000–2011. (B) Predicted surface area profiles for Site B (amino acid residues 293–395) did not show definitive profiles, and surface motif types could not be defined from this data.

### Nucleic Acid Extraction and Reverse Transcription

Total nucleic acid was extracted from a 200 µl aliquot of the suspension using the QIAxtractor automated nucleic acid extraction platform (QIAGEN Ltd., Crawley, UK) and the VX Reagent & Plasticware kit (QIAGEN). Complementary DNA (cDNA) was generated by reverse transcription; extracted nucleic acid was incubated in the presence of 50 pmol of poly(T) primer, Tris-HCl buffer (pH 8.3), 5 mM MgCl_2_, 1 mM each dNTP and 200U SuperScript-III reverse transcriptase (Life Technologies). The reverse transcription reaction was performed at 42°C in a water bath.

**Figure 4:**
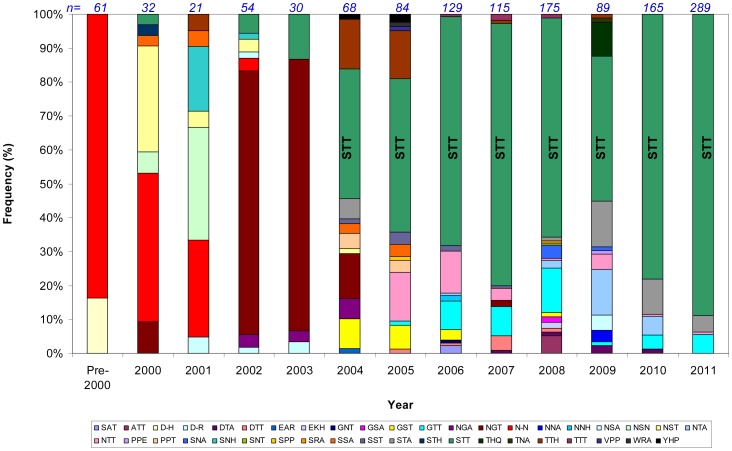
Frequencies of detection of Site B motifs 2000–2011. A single motif [STT] (green) was the most frequently detected throughout the analysis period. Between 2008 and 2009 the amount of diversity at Site B increased, with 15 and 13 different motifs detected, respectively. Amino acid motifs designated by standard IUPAC single-letter amino acid code.

### Amplification of P2 Domain

The NoV P2 domain was amplified by polymerase chain reaction (PCR) as previously described [15], [19]. For pyrosequencing, biotinylated amplicons were generated using a biotinylated reverse primer.

### Pyrosequencing

Pyrosequencing was performed using the QIAGEN PyroMark ID platform according to the manufacturer’s instructions. Briefly, biotinylated amplicons were immobilized on streptavidin coated sepharose beads, denatured and washed to generate single stranded (ss)DNA using the PyroMark Vacuum Prep Workstation (QIAGEN). The ssDNA was then released to the PSQ plate containing annealing buffer and sequencing primer, which was briefly heated then cooled to allow sequencing primer annealing. Pyrosequencing reactions were performed using PyroMark Gold Q96 SQA reagents and the PyroMark ID instrument (QIAGEN) with 100 de-novo nucleotide dispenses. Negative controls were also included in the runs. Resulting pyrograms were automatically analyzed using the PyroMark analysis software. Generated sequences were exported in FASTA format and analysed as described below.

The following pyrosequencing primers were designed for this study:

Pyro_Site A1∶3′-TCTGCTGTCAACATCTGC-5′

Pyro Site B1∶3′-AAAACACGAAATTCACCCCAGTCGGYGT-5′

Pyro_Site B2∶3′-CAAAACACGAAATTCACCCCAGY-5′

### Genbank Data & Sequence Bioinformatics

A total of 250 GII-4 NoV sequences available from Genbank were also included in the analysis. These were a mixture of full-length genome, full-length capsid (ORF2) and partial capsid sequences. A complete list of accession numbers for the samples included are provided in Table S1.

**Figure 5:**
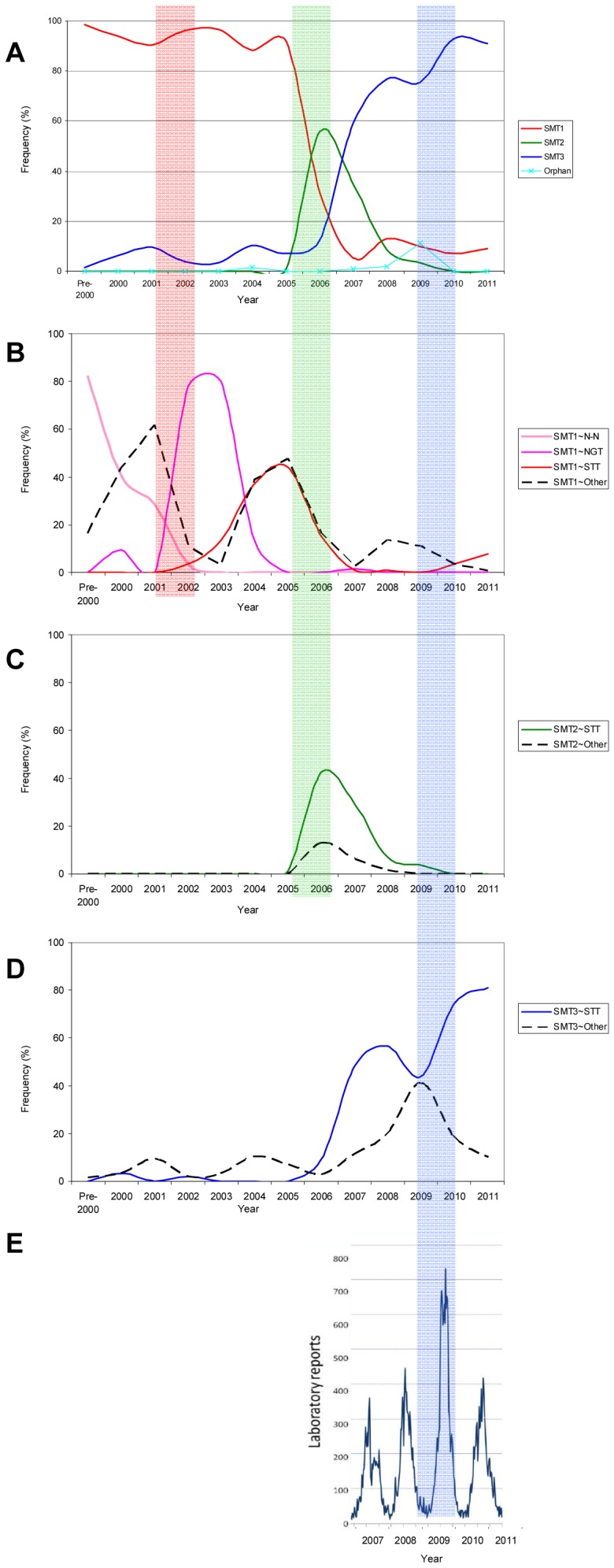
Higher-than-average NoV activity correlates with shift in detected Site A surface motif type and increased amino acid diversity at Site B. (A) Frequency of detected surface motif types (SMT) of Site A, indicated in red (SMT1), green (SMT2) and blue (SMT3) (as in Figure 3) changes over the time period analysed. (B) Frequency of SMT1 NoV strains with Site B motifs N-N (pink), NGT (purple), STT (red), and all other Site B amino acid motifs detected (dashed black). (C) Frequency of SMT2 NoV strains with Site B motif STT (green), and all other Site B amino acid motifs detected (dashed black). (D) Frequency of SMT3 NoV strains with Site B motif STT (blue), and all other Site B amino acid motifs detected (dashed black). Shaded columns highlight periods of high NoV activity in 2001/2002 (red), 2005/2006 (green) and 2009/2010 (blue). (E) Periods of high NoV are defined by the epidemiological trends observed over time. The data shown here presents epidemiological data (number of NoV-positive laboratory reports) for the period from 2007 to 2011 for England & Wales. Prior to 2007, the data is not comparable due to differences in reporting rates, and so is not shown. A clear increase in NoV-positive laboratory reports is seen in 2009/2010, indicating an epidemiologically-defined period of high NoV activity. Amino acid motifs designated by standard IUPAC single-letter amino acid code.

Where full-length P2 domain amplicons were generated and sequenced in place of pyrosequencing data, sequence analysis was performed using Bionumerics v6.1 (Applied Maths, Kortijk, Belgium). Amino acid sequences were deduced from nucleotide sequences using BioEdit [20].

Throughout, amino acid motifs are designated by standard IUPAC single-letter amino acid code.

### Homology Modelling

For homology modelling, programs SPDBV [21] and NOC 3.1 (http://noch.sourceforge.net/) were used to model amino acid changes at Site A (amino acid positions 296–298) and Site B (amino acid positions 393–395) within the P2 domain (Figure 1). Modelling used the published crystal structure of GII-4 NoV strain VA387 (PDB: 2OBS) [22] as a template. Changes to the predicted surface area profile of the P2 domain model were quantified (in Å^2^) and numerical values obtained were plotted.

**Figure 6:**
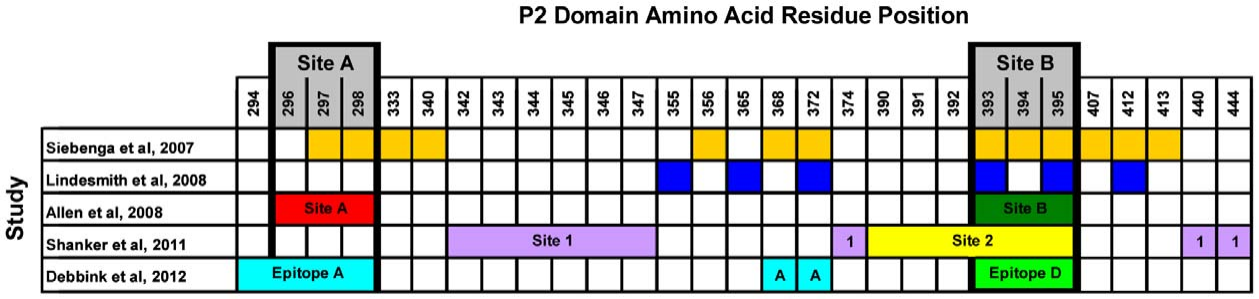
Key amino acid positions in the P2 domain, predicted to be involved in virus-host interactions. A summary of the data from 5 studies is presented in the table [14], [15], [16], [29], [31]. Key sites identified are predicted to modulate receptor binding interactions and/or the antigenic profile of the virus. In the study by Allen et al. [15], two sites were identified: Site A (red) and Site B (green). In the study by Shanker et al. [31], two sites were identified: Site 1 (purple) and Site 2 (yellow). In the study by Debbink et al. [29], two sites were identified: Epitope A (bright blue) and Epitope D (bright green). Site A and Site B as indicated at the top of the table (grey) are as defined in [14], [15], [16], [29], [31] and in this study.

## Results

In this study we determined the amino acid diversity at Site A and Site B in the P2 domain of GII-4 NoV strains (Figure 1) from 1062 clinical specimens associated with outbreaks of gastroenteritis in the UK during the period 2000–2011, and an additional 250 GII-4 NoV isolates from the period 1974–2009 available from Genbank (Table S1).

From the 1312 GII-4 NoV P2 domain sequences, we identified 23 unique Site A amino acid motifs and 41 unique Site B amino acid motifs, and these were found as 82 different Site A/B amino acid motif combinations. The frequencies of detection of each Site A/B amino acid motif combination are shown in Figure 2.

Only 5/82 (6%) of Site A/B motif combinations were detected with a frequency >5% (Figure 2). During the analysis period, 4 strain replacement events were observed where the Site A/B amino acid motif combination detected in more than 60% of isolates changed (Figure S1). The first replacement event occurred 2001–2002, when the motif combination [SHD∼N–N] was replaced almost entirely by [THN∼NGT]. This motif combination then dominated until 2004 when it was replaced by [TQN∼STT], and in 2006, this motif was succeeded by motif combination [TQE∼STT]. By 2008, [TQE∼STT] had been replaced by [SRN∼STT].

In order to understand the phenotypic characteristics of the Site A/B amino acid motifs detected, we used homology modelling to map these sites onto GII-4 NoV strain VA387 [22] and predict the effects of the detected Site A/B motif combinations on the virus structure around Site A and Site B. The data for predicted surface area profiles at each site were plotted to produce predicted surface area motifs (Figure 3).

Analysis of the amino acid motifs at Site A by homology modelling revealed that the majority (n = 19/23, 82.6%) belonged to one of three surface motif types (SMT-1, -2 and -3, Figure 3A). Some Site A amino acid motifs detected 2007–2009 could not be allocated into a specific SMT group (orphan motifs, n = 4).

Amino acid motif diversity was higher at Site B than at Site A, and a similar analysis of the amino acid motifs at Site B by homology modelling did not identify any grouping of Site B amino acid motifs, and so no surface motif types could be defined for this site (Figure 3B). However, it was noted that among the 41 unique Site B amino acid motifs detected, the motif [STT] was the most frequently detected (785/1312, 60%), and was detected in >40% of samples from 2004 onwards (Figure 4).

In the period from 2000 to 2005, the majority of GII-4 NoV isolates had predominantly type-1 surface motifs (SMT1) (Figure 5). Type-2 surface motifs (SMT2) were first detected in 2005, and in 2006 these displaced strains with SMT1. This change in SMT coincided with a period of high NoV activity in 2005/2006. SMT2 declined in subsequent years, and by 2010 were no longer detected. Type-3 motifs (SMT3) had been detected with very low frequency before 2000, and did not become dominant until 2007. After 2007, SMT3 displaced SMT2 as the dominant Site A surface motif, and remained dominant until 2011 (Figure 5A).

Type-1 surface motifs (SMT1) were most commonly found in association with one of three Site B amino acid motifs, namely [N–N], [NGT] and [STT] (Figure 5B). Prior to 2002, [N–N] was most frequently detected in association with SMT1, then between 2002 and 2004 [N–N] was replaced by [NGT], and this motif was succeeded by [STT] between 2004 and 2007, when strains with SMT1 motifs became infrequently detected.

Both SMT2 and SMT3 strains were most commonly detected in association with the [STT] amino acid motif at Site B (Figure 5C & D, respectively). In 2009 the number of SMT3 strains detected in association with [STT] at Site B declined, and was accompanied by a concomitant increase in the diversity of the Site B amino acid motif associated with SMT3. This event coincided with a period of high NoV activity in the winter of 2009/2010.

## Discussion

We previously identified two sites (Site A and Site B) within the hypervariable P2 domain of the NoV major capsid protein VP1 that undergo selective amino acid changes that coincide with the appearance of epidemiologically significant GII-4 NoV strains in the population [15] Furthermore, these two sites were sufficient to define strain-specific GII-4 antibody-antigen interactions [18]. Our data, together with that of others contributes to a growing body of evidence that suggests novel GII-4 NoV antigenic variants emerge in response to selective pressure from the host population [14], [15], [16], [23], [24].

In this paper, we describe the characterisation of the amino acid motifs at Site A and Site B of 1312 GII-4 NoV strains; 1062 nucleotide sequences derived from strains collected in England & Wales in the period from 2000 to 2011, with greater number of samples available for testing from 2004 onwards, and 250 GII-4 NoV sequences available from Genbank collected between 1974 and 2009 (Table S1) were also included in the analysis.

We explored the value of modelling the predicted surface area derived from the different amino acid substitutions at sites A and B as predictors for significant changes that correlated with the emergence and/or switch of epidemic strains. During the analysis period, three years were associated with higher-than-average NoV activity in the UK: 2002, 2006 and 2009. This analysis showed that for Site A, three major surface area motif types (SMTs) could be defined over the analysis period. In contrast, the diversity at Site B could not be used to define SMTs in the same way as for Site A. Plotting the frequency of SMT associated with site A and the diversity associated with site B showed that years of higher-than-average NoV activity were associated with: (i) a switch in the most frequently detected Site A surface motif type, or (ii) an increase in the amino acid diversity observed at Site B (Figure 5).

Here, we propose a model of antigenic evolution for GII-4 NoV strains based on sequence diversity at both Site A and Site B, and epidemiological trends observed in between 2000 and 2011.

We predict that Site A is a major epitope on the virus surface, responsible for defining the antigenic profile of GII-4 NoV strains belonging to different neutral networks of viruses, described previously [15]. Although a significant degree of amino acid diversity is seen at Site A, most of the diversity seen is likely to be phenotypically neutral, as defined in terms of surface area, with the vast majority of strains belonging to only 3 distinct SMTs, termed 1, 2 or 3. Interestingly, the emergence of an SMT correlated with the epidemic waves, and was accompanied by displacement of the previously dominant SMT. Therefore SMTs at Site A define three different “epochs” of NoV activity between 2000 and 2011.

Although the diversity observed at site B could not be correlated with discreet SMTs, we observed periodic increases in amino acid motif diversity at this site, and this coincided with periods of high NoV activity (Figure 5). We predict that Site B is a minor epitope on the virus surface, and that diversity at this site is associated with maintaining sufficient antigenic diversity within the virus population allowing for the persistence of GII-4 strains in the pre-exposed human population between epidemic waves or NoV “epochs”. The level of diversity at Site B may influence the year-on-year epidemics observed in successive winter seasons, in contrast to Site A in which significant changes occur more sporadically.

The large NoV epidemic described worldwide in 2002 coincided with the emergence of a drastic change at Site B in the form of an amino acid insertion, in the context of strains of SMT1 at Site A. This significant change appears to clearly correlate with the pandemic event, but GII-4 strains of SMT1 continued to dominate until the 2005/6 winter epidemic, possibly though the diversity emerging at Site B. The epidemic in 2006 correlated with a switch to SMT2, which appeared to vanish more rapidly than SMTs 1 and 3, and this may be explained though the lower degree of diversity seen at the Site B associated with the strains of SMT2. Finally, although SMT3 had been circulating at relatively low levels for a decade, it did not cause a large epidemic wave until 2009, but only when it was found in association with the highest level of diversity at Site B.

This therefore suggests that both these sites contribute to immune evasion and hence the persistence of GII-4 strains in the human population. It is also noteworthy that although there are epidemic events which are described concomitantly worldwide, such as those in 2002 and 2006, which could be defined as NoV pandemics, there are other high NoV activity years that appear to be more localised. For example, an increase in NoV activity described in the Netherlands [25] and Australia [26] was not mirrored in the UK, whereas the increased NoV activity detected in the UK in 2009 was not seen in other countries [27]. These differences may be driven by the degree of diversity within Site B, which may be responsible also for more localised epidemics. In 2009, a new GII-4 strain, termed New Orleans, emerged across the USA [28]. This replaced the Minerva (2006b) GII-4 strain as the most prevalent NoV strain in the USA, however, surveillance did not report any increase in numbers of NoV outbreaks during the 2009/10 winter following emergence of the novel New Orleans strain [27], [28].

We compared both a Minerva (2006b)-like strain (Hu/OSD-CS/2006/USA, Accession Number: EU078417) and a 2009 New Orleans-like strain (Hu/GII.4/New Orleans 1805/2009/USA, Accession Number: GU445325) with our data set and found that both strains had the same surface motif; SMT3. Given that the population had recently been exposed to the Minerva (2006b) strain, the majority of the population would have been protected against the 2009 New Orleans strain that belonged to the same antigenic cluster. In fact, both Site A and Site B motifs in these strains were identical to each other, and to the dominant [SRN∼STT] strains detected in our analysis.

There is additional *in vitro* data supporting evidence for the model of evolution and immune evasion proposed here. We have previously demonstrated that variant-specific antigenic profiles of GII-4 NoV strains can be defined by the amino acid residues at Site A and Site B [18]. Mutation of Site A was sufficient to entirely abolish mAb recognition of its homologous VLP, whereas mutation at Site B resulted in a significant reduction in mAb-VLP recognition was observed, but not complete abolishment of binding [18].

Data from other studies also supports our conclusion that Site A and Site B have important roles in defining the antigenic profile of GII-4 NoVs. Of the ∼120 amino acids in the P2 domain, relatively few are likely to be involved in defining key epitopes, based on the work of a number of laboratories (Figure 6) [18], [29], [30], [31]. Comparison of the data from 5 different studies coincides in assigning residues around positions 296–298 (Site A) and 393–395 (Site B) as likely determinants of the antigenic phenotype of the virus (Figure 6).

Fine resolution epitope mapping by systematic mutation of the capsid protein revealed that residues comprising Site A, plus three others within the P2 domain (Epitope A) define a key surface epitope, and also that the residues comprising Site B (Epitope D) are involved in modulating interactions between virus and histo-blood group antigens (HBGAs) [29]. This is in agreement with data from Shanker et al. [31] who describe two sites (Site 1 and Site 2) in the P2 domain that influence the interactions between the virus and HBGAs. In their study, using crystallographic structural techniques, the authors found that conserved residues in the P2 domain (Site 1) were involved in a definitive binding interaction with conserved α-fucose residues among the Lewis HBGAs – in agreement with an earlier study by Cao et al. [22] – and that Site 2 (or Site B) was involved in stabilisation of the HBGA interaction through binding of the β-galactose residue in the HBGA molecule.

The data now available from different studies seems to suggest a major role for the residues in and around Site A in defining a dominant surface epitope [31]. The data presented here suggests surface/structure changes at Site A are more restricted, concurrent with the observation that these residues are involved in binding contacts with Lewis HBGA molecules [18], [29]. The structures defined by amino acid residues at Site A must be under pressure to maintain their receptor binding functions; therefore mutations that alter the antigenic profile at this site but maintain receptor binding functionality are likely to be successfully selected in the population of circulating strains.

Data for Site B suggests a more subtle role in terms of defining a surface epitope, but an important role in modulating the strength of binding interactions with HGBA molecules [31]. The data presented in this study is in agreement with these findings, as the residues at Site B show much more plasticity than those at Site A. Furthermore, successful Site A motifs are associated with a diverse array of Site B motifs, suggesting that the virus uses the genetic and antigenic flexibility at Site B to enhance and modulate interactions with receptors and the immune system occurring at Site A.

## Supporting Information

Figure S1 **Observed GII-4 norovirus strain replacement events.** The figure shows the frequencies of detection in each year for the five most commonly detected Site A/B motif combinations (Figure 2). Strain replacement events (where the most frequently detected Site A/B motif combination in that year was different from that in the previous year) are indicated above the graph by arrows. We observed four strain replacement events between 2001–2002, 2003–2004, 2005–2006 & 2007–2008.
(TIF)

Table S1 **List of accession numbers for GII-4 norovirus strains used in this study.** A total of 250 GII-4 NoV sequences from strains isolated 1974–2009 that were available from Genbank were included in our analysis. These were a mixture of full-length genome, full-length capsid (ORF2) and partial capsid sequences.
(PPT)

## References

[1] Tam CC, Rodrigues LC, Viviani L, Dodds JP, Evans MR, et al (2011) Longitudinal study of infectious intestinal disease in the UK (IID2 study): incidence in the community and presenting to general practice. Gut (2012) 61(1): 69–77. 10.1136/gut.2011.238386 PMC3230829 21708822

[2] Hutson AM, Atmar RL, Estes MK (2004) Norovirus disease: changing epidemiology and host susceptibility factors. Trends Microbiol 12: 279–287. 15165606 10.1016/j.tim.2004.04.005 PMC7172956

[3] Harris JP, Edmunds WJ, Pebody R, Brown DW, Lopman BA (2008) Deaths from norovirus among the elderly, England and Wales. Emerg Infect Dis 14: 1546–1552. 18826817 10.3201/eid1410.080188 PMC2609872

[4] Mattner F, Sohr D, Heim A, Gastmeier P, Vennema H, et al (2006) Risk groups for clinical complications of norovirus infections: an outbreak investigation. Clin Microbiol Infect 12: 69–74. 16460549 10.1111/j.1469-0691.2005.01299.x

[5] Wingfield T, Gallimore CI, Xerry J, Gray JJ, Klapper P, et al (2010) Chronic norovirus infection in an HIV-positive patient with persistent diarrhoea: a novel cause. J Clin Virol 49: 219–222. 20863753 10.1016/j.jcv.2010.07.025

[6] Zheng DP, Ando T, Fankhauser RL, Beard RS, Glass RI, et al (2006) Norovirus classification and proposed strain nomenclature. Virology 346: 312–323. 16343580 10.1016/j.virol.2005.11.015

[7] Teunis PF, Moe CL, Liu P, Miller SE, Lindesmith L, et al (2008) Norwalk virus: how infectious is it? J Med Virol 80: 1468–1476. 18551613 10.1002/jmv.21237

[8] Matsui SM, Greenberg HB (2000) Immunity to Calicivirus Infection. J Infect Dis 181: S331–S335. 10804146 10.1086/315587

[9] Johnson PC, Mathewson JJ, DuPont HL, Greenberg HB (1990) Multiple-challenge study of host susceptibility to Norwalk gastroenteritis in US adults. J Infect Dis 161: 18–21. 2153184 10.1093/infdis/161.1.18

[10] Koopman JS, Eckert EA, Greenberg HB, Strohm BC, Isaacson RE, et al (1982) Norwalk virus enteric illness acquired by swimming exposure. Am J Epidemiology 115: 173–177. 10.1093/oxfordjournals.aje.a113288 6277185

[11] Tan M, Jiang X (2005) Norovirus and its histo-blood group antigen receptors: an answer to a historical puzzle. Trends Microbiol 13: 285–293. 15936661 10.1016/j.tim.2005.04.004

[12] Tan M, Jiang X (2008) Association of histo-blood group antigens with susceptibility to norovirus infection may be strain-specific rather than genogroup dependent. J Infect Dis 198: 940–941. 10.1086/589810 18721066

[13] Donaldson EF, Lindesmith LC, Lobue AD, Baric RS (2010) Viral shape-shifting: norovirus evasion of the human immune system. Nat Rev Microbiol 8: 231–241. 20125087 10.1038/nrmicro2296 PMC7097584

[14] Lindesmith LC, Donaldson EF, Lobue AD, Cannon JL, Zheng DP, et al (2008) Mechanisms of GII.4 Norovirus Persistence in Human Populations. PLoS Med 5: e31. 18271619 10.1371/journal.pmed.0050031 PMC2235898

[15] Allen DJ, Gray JJ, Gallimore CI, Xerry J, Iturriza Gomara M (2008) Analysis of Amino Acid Variation in the P2 Domain of Norovirus VP1 Protein Reveals Putative Variant-Specific Epitopes. PLoS One 3: 1–9. 10.1371/journal.pone.0001485 PMC2194622 18213393

[16] Siebenga JJ, Vennema H, Renckens B, de Bruin E, van der Veer B, et al (2007) Epochal Evolution of GGII.4 Norovirus Capsid Proteins from 1995 to 2006. J Virol 81: 9932–9941. 17609280 10.1128/JVI.00674-07 PMC2045401

[17] Lopman B, Vennema H, Kohli E, Pothier P, Sanchez A, et al (2004) Increase in viral gastroenteritis outbreaks in Europe and epidemic spread of new norovirus variant. Lancet 363: 682–688. 15001325 10.1016/S0140-6736(04)15641-9

[18] Allen DJ, Noad R, Samuel D, Gray JJ, Roy P, et al (2009) Characterisation of a GII-4 norovirus variant-specific surface-exposed site involved in antibody binding. Virology Journal 6: 150. 19781066 10.1186/1743-422X-6-150 PMC2762976

[19] Xerry J, Gallimore CI, Iturriza Gomara M, Allen DJ, Gray JJ (2008) Transmission Events Within Outbreaks of Gastroenteritis Determined Through the Analysis of Nucleotide Sequences of the P2 Domain of Genogroup II Noroviruses J Clin Microbiol. 46: 947–953. 10.1128/JCM.02240-07 PMC2268335 18216210

[20] Hall TA (1999) BioEdit: a user-friendly biological sequence alignment editor and analysis program for Windows 95/98/NT. Nucl Acids Symp Ser 41: 95–98.

[21] Guex N, Peitsch MC (1997) SWISS-MODEL and the Swiss-PdbViewer: an environment for comparative protein modeling. Electrophoresis 18: 2714–2723. 9504803 10.1002/elps.1150181505

[22] Cao S, Lou Z, Tan M, Chen Y, Liu Y, et al (2007) Structural Basis for the Recognition of Blood Group Trisaccharides by Norovirus. J Virol. 81(11): 5949–57. 10.1128/JVI.00219-07 PMC1900264 17392366

[23] Lindesmith LC, Donaldson EF, Baric RS (2011) Norovirus GII.4 Strain Antigenic Variation. J Virol 85: 231–242. 20980508 10.1128/JVI.01364-10 PMC3014165

[24] Bok K, Abente EJ, Realpe-Quintero M, Mitra T, Sosnovtsev SV, et al (2009) Evolutionary dynamics of GII.4 noroviruses over a 34-year period. J Virol 83: 11890–11901. 19759138 10.1128/JVI.00864-09 PMC2772697

[25] Kroneman A, Vennema H, van Duijnhoven YT, Duizer E, Koopmans M (2004) High number of norovirus outbreaks associated with a GGII.4 variant in the Netherlands and elsewhere: does this herald a worldwide increase? EuroSurveillance 8.

[26] Bull RA, Tu ET, McIver CJ, Rawlinson WD, White PA (2006) Emergence of a New Norovirus Genotype II.4 Variant Associated with Global Outbreaks of Gastroenteritis. J Clin Microbiol 44: 327–333. 16455879 10.1128/JCM.44.2.327-333.2006 PMC1392656

[27] Yen C, Wikswo ME, Lopman BA, Vinje J, Parashar UD, et al (2011) Impact of an emergent norovirus variant in 2009 on norovirus outbreak activity in the United States. Clin Infect Dis 53: 568–571. 21832262 10.1093/cid/cir478

[28] Vega E, Barclay L, Gregoricus N, Williams K, Lee D, et al (2011) Novel surveillance network for norovirus gastroenteritis outbreaks, United States. Emerg Infect Dis 17: 1389–1395. 21801614 10.3201/eid1708.101837 PMC3381557

[29] Debbink K, Donaldson EF, Lindesmith LC, Baric RS (2012) Genetic Mapping of a Highly Variable Norovirus GII.4 Blockade Epitope: Potential Role in Escape from Human Herd Immunity. J Virol 86: 1214–1226. 22090110 10.1128/JVI.06189-11 PMC3255819

[30] Siebenga JJ, Lemey P, Kosakovsky Pond SL, Rambaut A, Vennema H, et al (2010) Phylodynamic reconstruction reveals norovirus GII.4 epidemic expansions and their molecular determinants. PLoS Pathog 6: e1000884. 20463813 10.1371/journal.ppat.1000884 PMC2865530

[31] Shanker S, Choi JM, Sankaran B, Atmar RL, Estes MK, et al (2011) Structural Analysis of Histo-Blood Group Antigen Binding Specificity in a Norovirus GII.4 Epidemic Variant: Implications for Epochal Evolution. J Virol 85: 8635–8645. 21715503 10.1128/JVI.00848-11 PMC3165782

