# Supplementary figures and images for: Molecular Evolution of GII-4 Norovirus Strains
Source: PLoS One. 2012 Jul 26;7(7):e41625. doi: 10.1371/journal.pone.0041625 (PMC3406047; doi:10.1371/journal.pone.0041625)

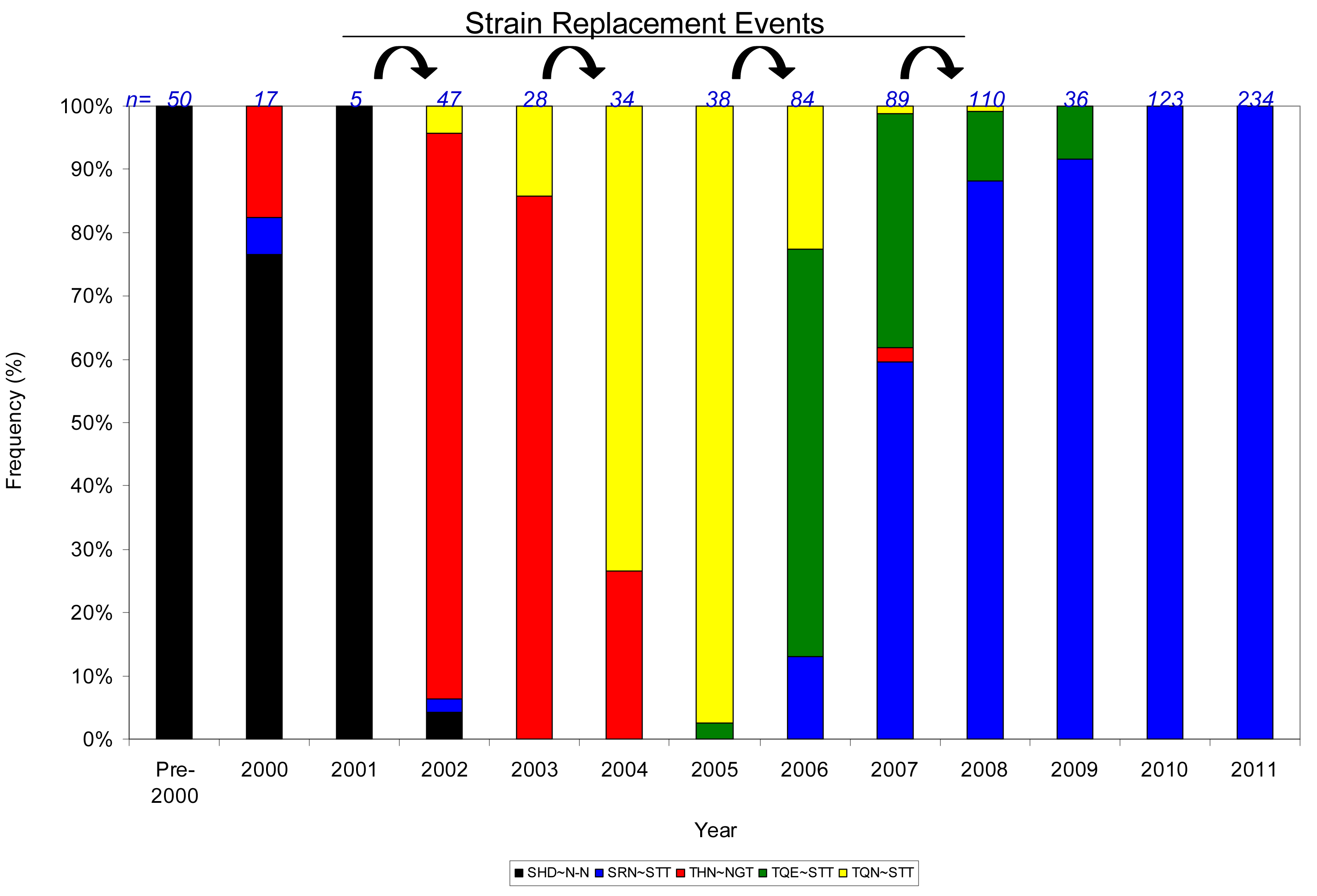

Supplement: Figure S1 — Observed GII-4 norovirus strain replacement events. The figure shows the frequencies of detection in each year for the five most commonly detected Site A/B motif combinations (Figure 2). Strain replacement events (where the most frequently detected Site A/B motif combination in that year was different from that in the previous year) are indicated above the graph by arrows. We observed four strain replacement events between 2001–2002, 2003–2004, 2005–2006 & 2007–2008. (TIF) [file pone.0041625.s001.tif]

## Slide 1
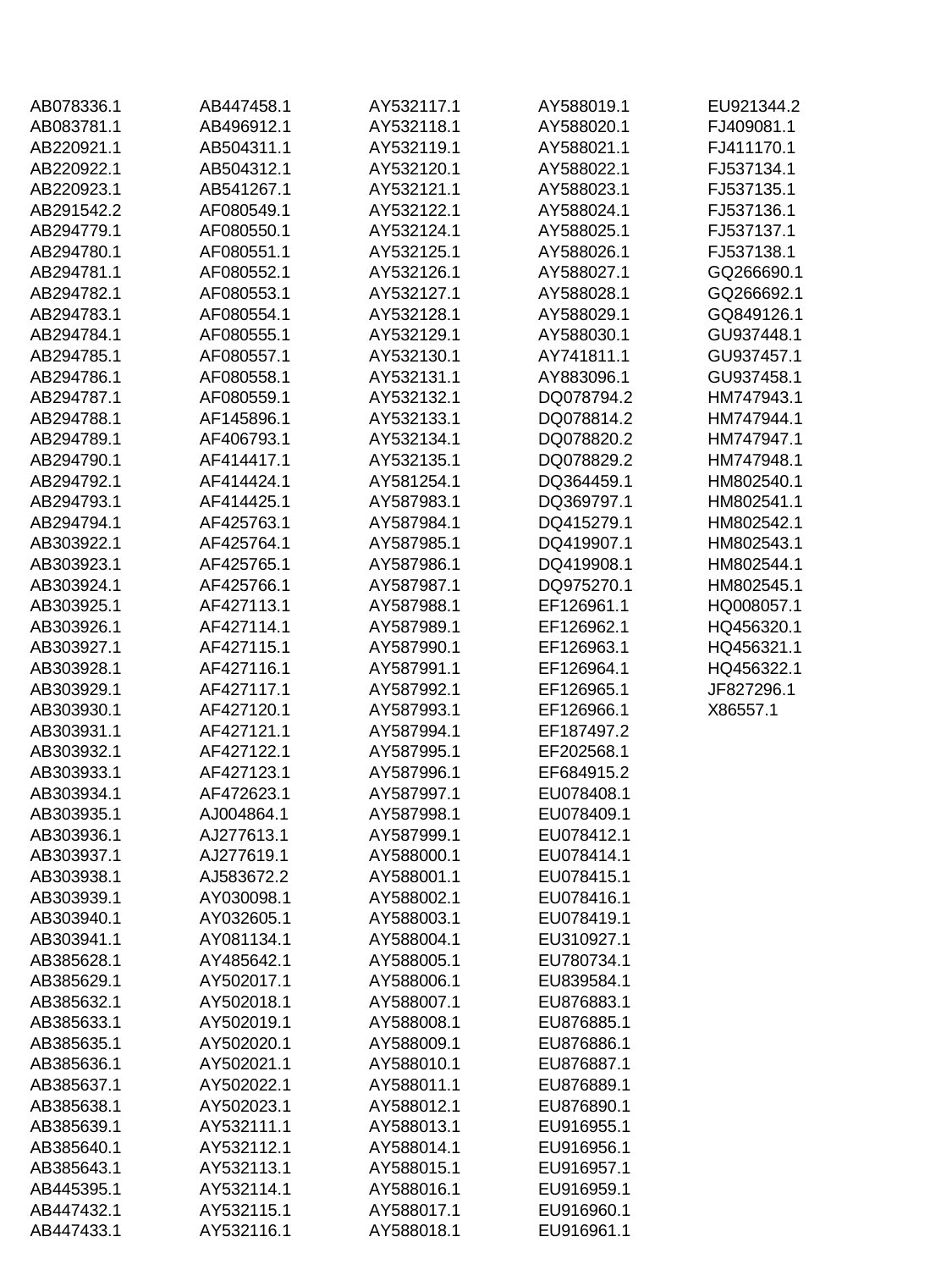

Supplement: Table S1 — List of accession numbers for GII-4 norovirus strains used in this study. A total of 250 GII-4 NoV sequences from strains isolated 1974–2009 that were available from Genbank were included in our analysis. These were a mixture of full-length genome, full-length capsid (ORF2) and partial capsid sequences. (PPT) [file pone.0041625.s002.ppt]
